# The Vapor Phase of Selected Essential Oils and Their Antifungal Activity *In Vitro* and *In Situ* against *Penicillium commune*, a Common Contaminant of Cheese

**DOI:** 10.3390/foods11213517

**Published:** 2022-11-04

**Authors:** Miroslava Hlebová, Denisa Foltinová, Dominika Vešelényiová, Juraj Medo, Zuzana Šramková, Dana Tančinová, Michaela Mrkvová, Lukáš Hleba

**Affiliations:** 1 Department of Biology, Faculty of Natural Sciences, University of SS. Cyril and Methodius, Nám. J. Herdu 2, SK-91701 Trnava, Slovakia; 2 Department of Microbiology, Faculty of Biotechnology and Food Sciences, Slovak University of Agriculture in Nitra, Tr. A. Hlinku 2, SK-94976 Nitra, Slovakia

**Keywords:** *Penicillium commune*, cyclopiazonic acid, essential oils, antifungal activity, cheese samples, sensory evaluation, vapor contact

## Abstract

This study aimed to determine the *in vitro* and *in situ* antifungal activity of (14) selected essential oils (EOS), namely clove, thyme, red thyme, litsea, eucalyptus, niaouli, fennel, anise, cumin, basil, rosemary, sage, bergamot mint, and marjoram, by vapor contact against the growth of two strains of *Penicillium commune* (KMi–183 and KMi–402). Furthermore, to exclude the negative effect of EOs on the lactic acid bacteria (LABs) (*Streptococcus* spp.) on cheeses, their influence was monitored. Next, the sensory evaluation of cheese treated by EOs was evaluated. The results show that litsea and clove EOs were the most effective in the vapor phase against both tested strains. These EOs were characterized by the highest amount of α- (40.00%) and β-Citral (34.35%) in litsea and eugenol (85.23%) in clove. The antitoxicogenic activity of less effective (in growth inhibition) EOs on cyclopiazonic acid (CPA) production by the tested strains was also observed. The growth of *Streptococcus* spp. (ranging from 8.11 to 9.69 log CFU/g) was not affected by the EOs in treated cheese. Even though the evaluators recognized some EOs in sensory evaluation by the triangle test, they did not have a negative effect on the taste and smell of the treated cheeses and were evaluated as edible. The antifungal activity of EOs against several types of microscopic fungi and their effect on the sensory properties of treated foods needs to be further tested to achieve the most effective protection of foods from their direct contaminants.

## 1. Introduction

Dairy products are less susceptible to contamination by various microorganisms mainly because they are kept in the cold, contain organic acids, have an acidic pH, or are fermented. However, despite this, there are many spoilage microorganisms that can survive or grow on dairy products, especially on cheese. Mainly fungi belong to these microorganisms. Fungi are a significant cause of food spoilage due to their great versatility for growing on substrates and in conditions where other microorganisms cannot grow [1]. Cheese spoilage by fungi is a problem associated with quality reduction due to visible or invisible undesirable changes such as an unpleasant odor or flavor [2]. Some of the microscopic fungi growing on dairy products may also produce secondary metabolites—mycotoxins—hence, constituting a potential risk to food safety and, therefore, to human health [3]. The fungal genus most frequently isolated from spoiled cheese that regularly dominates in many studies is *Penicillium*, specifically, the *Penicillium commune* species (42%). *P. commune* can synthesize cyclopiazonic acid (CPA) [4]. CPA is not considered a potent acute toxin. However, chronic exposure to CPA can cause degenerative effects (nephrotoxic, neurotoxic, etc.), and its occurrence in food should not be underestimated [5].

Currently, various methods are used to treat food, such as heat treatment, reduction of water activity, various packaging methods, irradiation, and synthetic preservatives. However, these substances are slowly starting to be replaced by natural ones because consumers are looking for healthier foods. Various natural antioxidants, antimicrobial substances, sweeteners, and dyes originating from plants are used in food to protect it from spoilage microorganisms [6,7]. In addition, essential oils (EOs) or their components, as natural antimicrobial agents, could protect against microbial damage to the cheese and thus prolong its storability [8]. The antimicrobial and antioxidant properties of EOs against a wide range of microorganisms have already been confirmed. Due to their hydrophobicity, individual components of EOs easily pass through prokaryotic or eukaryotic cell membranes, interfering with molecular transport mechanisms and leading to cell lysis [9]. Some EOs can even inhibit the production of secondary metabolites, mycotoxins [10,11]. Laird and Phillips [12] compared studies of the antimicrobial and antifungal activity of EOs in the vapor and liquid phases and indicated that the use of the EO vapors seems to be more effective. Inouye et al. [13] indicated that the reason why the vapor phase is more effective could be that the lipophilic components of the EOs react with the aqueous components of the nutrient medium (micelles are formed) and this prevents the attachment of the EOs to the microorganism. EO vapors, on the other hand, can freely attach to the microorganism. Moreover, the vapor phase does not affect the sensory properties of food, accordingly, as the EO liquids do. However, the applied concentration of EOs should be considered carefully because the aromatic properties of these substances are still intense and could have different interactions on various food matrices [14]. Therefore, they are suitable for use in the food industry as preservatives. Their application in food is regulated by The European Commission in Europe and by the Food and Drug Administration (FDA) in the United States. EOs are generally recognized as safe (GRAS) by the FDA, which makes them ideal antimicrobial agents and preservatives [15].

For this reason, it would be possible to use EOs as effective preservatives to protect dairy products from the microbial spoilage production of secondary metabolites and to extend their shelf life [16]. EOs and their effects directly on cheeses have already been tested by several authors, for example, by direct addition to the cheese during its preparation [17], encapsulation, and subsequent application to cheeses [18], or by using films or coatings on the surface of cheeses [19]. However, the effect of the vapor phase of EOs on the growth of spoilage fungi, the production of their mycotoxins, and their influence on the organoleptic properties of cheeses directly in the *in situ* conditions (on the cheese samples) have not been investigated yet.

Therefore, this study aimed to investigate the antifungal effect of selected EOs on the growth of the important cheese contaminant *P. commune* (two strains) using the vapor phase in *in vitro* and *in situ* conditions (directly on model food). Furthermore, in this study, the effect of EOs in the vapor phase on the production of cyclopiazonic acid (*in vitro*) by the tested strains was evaluated, and their effect on the sensory properties of the tested cheeses (*in situ*).

## 2. Materials and Methods

### 2.1. Essential Oils Samples

The fourteen EOs—clove (*Syzygnium aromaticum* L.), thyme (*Thymus vulgaris* L.), red thyme (*Thymus serpyllum coccineus* L.), litsea (*Litsea cubeba* Lour. Per.), eucalyptus (*Eucalyptus globulus* Labill), niaouli (*Melaleuca quinquenervia* b.s. viridiflorol), fennel (*Foeniculum vulgare* L.), anise (*Pimpinella anisum* L.), cumin (*Carum carvi* L.), basil (*Ocinum basilicum* L.), rosemary (*Rosmarinus officinalis* L.), sage (*Salvia officinalis* L.), bergamot mint (*Mentha citrate* L.), and marjoram (*Origanum majorana* L.)—were obtained from commercial supplier Calendula a.s. (Nová Ľubovna, Slovakia) by hydrodistillation. Obtained samples were stored hermetically in sealed flasks at 4 °C in the dark until *in vitro* and *in situ* analysis. Gas Chromatography–Mass Spectrometry (GC–MS) with the following flame ionization detector (GC–FID) for chemical composition and relative proportions of EO constituents were used. The methods used are detailed in a previous study [11]. Authentication of the relative proportion of the EO constituents was confirmed by comparing their spectra with authentic standards (Sigma–Aldrich, Munich, Germany).

#### 2.1.1. Fungal Strains

In this experiment, two strains of *Penicillium commune* (KMi–183 and KMi–402) were used. Both tested strains were previously isolated and collected from commercial cheese products (45% Edam cheese). The strains were cultivated on CYA (Czapek yeast extract agar) (CYA, HiMedia, Mumbai, India) at 25 ± 1 °C for 7 days in the dark before analysis. The *Penicillium* isolates were identified by ITS rDNA sequencing [20,21]. The following accession numbers were generated by the GenBank database, where obtained sequences were deposited: OP439736 (*P. commune* KMi–183) and OP439737 (*P. commune* KMi–402).

#### 2.1.2. *In Vitro* Antifungal Analysis

A modified gas diffusion method for the antifungal activity of selected EOs was performed. The method is detailed in Císarová et al. [10]. Saboraud dextrose agar (SDA, HiMedia, Mumbai, India) for antifungal activity testing was used in this study. At the beginning of this experiment, the highest concentration of EOs (500 μL/L of air) was tested. The EOs were applied using a micropipette onto the filter paper disks (d = 30 mm) placed on the inner surface of the lids. The Petri dishes (PDs) divided into three sectors were used in independent repetitions (*n* = 6). Each sector of the PD contained 5 mL of SDA medium. After preparation and inoculation (in the center of each sector of PDs) with fungal strains, the Petri dishes were tightly sealed with parafilm and cultivated. The cultivation of *Penicillium* strains was performed in two ways. Diameters of fungal colonies and their growth were measured on the 3rd, 7th, 11th, and 14th day of incubation in two perpendicular directions at 25 ± 1 °C. Parallel cultivation of fungal isolates with EOs was done at a temperature of 5 ± 1 °C in a refrigerator for 35 days and diameters of fungal colonies were measured on the 3rd, 7th, 11th, 14th, 21st, 28th, and 35th day. The percentage of mycelial growth inhibition after all cultivation days was calculated according to the following formula:

Mycelial growth inhibition (MGI %) = [(dc − dt)/dc] × 100

where dc = average (mm) increases in mycelial growth in control, and dt = average (mm) increases in mycelial growth in treatment. Then, the minimum inhibitory doses (MIDs) of the most potent EOs were tested in the same ways as described above (PDs divided into three sectors in two repetitions were used (*n* = 6)). A concentration range between 15.62 and 250 μL/L of air was used. All EOs were dissolved in ethyl acetate. The MID was defined as the lowest concentration of the EO that caused any visible growth after 14 days of cultivation in comparison with the control.

#### 2.1.3. Cyclopiazonic Acid (CPA) Analysis

The inhibition of cyclopiazonic acid production by EOs was determined by the qualitative screening TCL method. The method is described in previous studies [22,23]. The inhibition of CPA by EOs was tested after 14 and 35 days of *Penicillium commune* cultivation after treatment with EOs only at the highest tested concentration of 500 µL/L of air. *Penicillium* strains were cultivated on Czapek yeast extract agar (CYA, HiMedia, Mumbai, India) at 25 ± 1 °C for 14 days and at 5 ± 1 °C for 35 days and treated by EOs (500 μL/L of air) in the same way as described in Section 2.1.2. Only strains that showed visible growth in treatment with EOs and control samples were used for analysis. One or two small pieces (each 5 × 5 mm) (the number of pieces depended on how large the colony had grown but could not be smaller than 1 cm) were cut from the growing colony on CYA plates and placed into 1.5 mL microtubes. Then, 500 mL of extraction solvent (chloroform:methanol, 2:1, *v*/*v*) was added to the microtubes containing the agar plugs and shaken on a vortex for at least 2 min. Subsequently, 30–50 µL of the obtained extracts were applied as spots on the TLC plate (Silicagel 60, Merck, Munich, Germany) 1 cm apart. Consequently, the spots were dried, and the plates were developed in a toluene:ethylacetate: formic acid (6:3:1, *v*/*v*/*v*) solvent system that gave an average R_f_ value of 0.65 for CPA. CPA was visualized as a violet tailing spot visible at daylight after applying Ehrlich’s reagent (Sigma–Aldrich, Munich, Germany) and heating the chromatographic plate to 130 °C for 8 min. The authentic standard of CPA (Sigma–Aldrich, Munich, Germany) as a positive control was used.

### 2.2. In Situ Antifungal Analysis on Cheese

#### 2.2.1. Cheese Inoculation and Essential Oil Treatment

The *in situ* experiments on cheese samples were performed according to Císarová et al. [10]. The cheese samples (45% Edam cheese obtained from different markets in Slovakia) with no signs of damage on the surface were cut into slices (approximately 50 g). The cheese slices were placed into 0.5 L sterile glass jars with a diameter of 115 mm and height 95 mm with arch cap and rubber seal (Bromioli Rocco, Fidenza, Italy). Fungal spore suspension of both *Penicillium* strains (final concentration of spores 1 × 10^6^ spores/mL) was prepared according to Božik et al. [24] by adjusting the density of suspension to 0.8 McFarland units. Subsequently, a 5 μL of the inoculum was added on top of the cheese slices at four different places. EOs were selected according to their best activity (where no fungal growth was detected) in evaluation of MIDs. Other ineffective EOs were not forwarded to in situ evaluation. Then, a 100 μL of each solution (EOs dissolved in ethyl acetate) was evenly distributed on a sterile paper filter disc (60 mm). The paper filter disc impregnated with EOs was inserted into the cover of the jar. The control group did not receive any EO treatment. Jars were hermetically closed and kept at refrigerator temperature (5 ± 1 °C) for 35 days in the dark. After 14 and 35 days of storage, the colonies with visible mycelial growth were counted.

#### 2.2.2. Essential Oil Effect on Lactic Acid Bacterial Vitality

In order to verify the possible negative effect of the EOs on the starter bacteria used in cheese production, all *in situ* tested samples were analyzed for lactic acid bacterial vitality. In this study, *Streptococcus* sp. as a starter culture for this type of cheese production was focused on and analyzed. Samples of *Streptococcus* sp. were obtained from the core and surfaces of cheese samples after 14 and 35 days of cultivation with EOs (250, 125 a 62.5 μL/L). Subsequently, all samples were homogenized by Heidolph DIAX 900 (Heidolph, Schwabach, Germany) and transferred to physiological solution (45 mL). Then, a decimal dilution system was used to prepare the following dilutions: 10^−5^, 10^−6^, and 10^−7^ spores/mL. Prepared inoculum (1 mL) was transferred to Petri dishes with M17 agar (containing sodium beta-glycerol and phosphate pentahydrate) (HiMedia, Mumbai, India) in triplicate. A cheese sample without EO treatment was used as a positive control. Agar plates were incubated at 37 ± 1 °C during 72 h. After incubation, grown colonies of lactic acid bacteria were counted and recalculated as log CFU/g.

#### 2.2.3. Sensory Evaluation of Treated Cheese

To evaluate the effect of EOs on the sensory properties of the cheese, EOs that showed complete (100%) antifungal activity in the previous experiments (*in vitro* and *in situ*), regardless of the cultivation days and temperature, were selected to treat the model food (cheese—Edam 45%). Approximately 100 g of non–inoculated cheese samples was cut into small cubes (1 × 1 cm) and then placed in sterile glass cups (same glass cups as used in *in situ* analysis) and treated with three concentrations (62.5, 125, and 250 μL/L of air) of the most effective EOs, as described in Section 2.2.1 The control sets were prepared with distilled water. The glasses prepared in this way were stored in a refrigerator at a temperature of 5 ± 1 °C. Sensory evaluation of cheese samples treated with EOs was performed after 14 and 35 days of storage in the cold. Then the triangle tests (two-tail) were used to evaluate differences between sensory characteristics of treated and untreated (control) samples according to ISO Standards 4120 [25]. Fifteen untrained assessors participated in the sensory evaluation. Each assessor received three coded samples each time (two from the same treatment and one without treatment). The assessors had to choose samples that differed from the other two in order to assess the acceptability of the taste and aroma of the treated cheeses. Water was used to neutralize the taste, and roasted coffee beans were used to neutralize the smell of cheese samples.

### 2.3. Statistical Evaluation

All experiments in this study were carried out in independent duplicate (*in vitro* analysis using the PDs divided into three sectors) and independent triplicate (*in situ* analysis). The results were obtained using SAS (one-factorial variance analysis and multifactorial variance analysis ANOVA 16.1 statistical program—analysis of variance (ANOVA)—and Tukey HSD 95% multiple range test were performed at significance level of *p* < 0.05). The results (obtained from *in vitro* testing the antifungal activity of EOs at a concentration 500 µL/L) were expressed as the mean of colony diameter for tested fungal strains ± the standard deviation (SD). The probity analysis was used for calculating MID_50_ and MID_90_ values (doses of EOs able to inhibit the growth to 50% and 90%, respectively). Triangle tests (two-tail test) were performed according to ISO 4120:2004 [25]. If the number of correct answers was greater than or equal to the value in the consulting tables (ISO 4120:2004) between the samples, a difference was detected. The obtained data were evaluated using the chi-squared binomial distribution with α-risk = 0.05 [26].

## 3. Results and Discussion

### 3.1. In Vitro Inhibitory Effect of Essential Oils on P. commune Strain Growth and MID Evaluation

The primary environment of *P. commune* is food, especially cheese, in which it is one of the most important contaminants, both in the finished products and in the process of their production [27]. Moreover, *P. commune* can produce cyclopiazonic acid. Therefore, it is necessary to look for possible ways to prevent its growth in food [28]. Thus, in this study, the antifungal effect of the 14 EOs was evaluated using the gas diffusion method against two *Penicillium commune* strains isolated from cheese. Firstly, to verify the antifungal effect of the used EOs, a higher concentration of 500 μL/L of air was used. Antifungal activity was tested at two cultivation temperatures of 25 ± 1 °C (cultivation for 14 days) and 5 ± 1 °C (cultivation for 35 days). The results (Table 1) show that EOs which were able to inhibit the growth of *P. commune* strains (KMi–183 and KMi–402) at both cultivation temperatures (5 ± 1 °C and 25 ± 1 °C) altogether included clove, red thyme, thyme, and litsea EOs.

Similar results to these EOs have been reported in previous studies against fungal species from the genus *Aspergillus* [10,11,21,29] and against some species from the genus *Penicillium* [22,23,30]. The growth of *P. commune* strain KMi–402 was also entirely inhibited by cumin and marjoram EOs after 14 days of cultivation at 25 °C and by fennel, anise, cumin, sage, and marjoram after 35 days of cultivation at 5 °C too. The growth of strain *P. commune* KMi–183 was inhibited entirely after 35 days of cultivation at 5 °C by anise, cumin, and marjoram EOs. Therefore, anise, cumin, and marjoram EOs showed 100% growth inhibition of both *P. commune* strains only at a lower temperature (5 ± 1 °C) during the 35 days of cultivation. In this study, *P. commune* strain KMi–183 was more resistant to the treatment by EOs at both used cultivation temperatures (25 ± 1 °C, cultivation for 14 days and 5 ± 1 °C, cultivation for 35 days) compared to control sets and the second strain KMi–403. EOs which were able to inhibit mycelial growth of *P. commune* strain KMi–183 with a very good inhibitory effect (over 80%) at 25 ± 1 °C included only cumin (87.46%) and marjoram (81.71%). In addition, other authors confirmed the good, but not better, antifungal activity of cumin EO against fungal species of *Aspergillus flavus* [31,32] and some fungal species of the genus *Penicillium* [33], and antifungal activity of marjoram EO against *Candida albicans* [34]. Eucalyptus and bergamot mint EOs showed the lowest inhibitory effect. In our study, the bergamot mint EO was characterized by the highest proportion of linalool (37.2%) and geraniol (42.1%). Other authors have also reported that EOs with a higher content of these components have a stimulating or no inhibitory effect on fungal growth [35,36]. The growth of *P. commune* strains was inhibited by bergamot mint after the 14th day of cultivation only with an MGI of 17.30% (strain KMi–183) and 33.67% (strain Kmi–402) and after the 35th day of cultivation with an MGI of 4.51% (strain KMi–183) and 11.77% (strain KMi–402). Despite the negative results obtained in treating *P. commune* strains with bergamot mint EO in our study, its antifungal activity has been reported in a study by Jakowienko et al. [37]. However, they used a disc diffusion method for testing the antifungal activity. However, Fazal et al. [38] state that EO from *Mentha citrata* L. is characterized by excellent antibacterial activity. In treatment with eucalyptus EO, strains of *P. commune* started to grow already on the third day of cultivation with an MGI after all cultivation periods of 2.80% (25 ± 1 °C) and 16.08% (5 ± 1 °C) for strain KMi–183 and 20.39% (25 ± 1 °C) and 36.34% (5 ± 1 °C) for strain KMi–402. The results are comparable with Davari and Ezazi [39], who tested the antifungal activity of several EOs, including eucalyptus EO, against the growth of important phytopathogenic fungi. They found that eucalyptus EO showed either a moderate or very weak inhibitory effect on the growth of these fungal species. The weak or moderate inhibitory effect of eucalyptus EO has also been demonstrated by Schroder et al. [40]. On the contrary, the very good antifungal activity of eucalyptus EO has been demonstrated in the work of other authors, Umereweneza et al. [41], but against different fungal species: *R. nigricans*, *A. flavus*, *A. niger*, *A. parasiticus*, *F. oxysporum*, and *P. digitatum*. However, in their research, the authors did not use the vapor phase of EOs but tested its antifungal activity in a contact form using the microdilution method.

Based on the *in vitro* analysis of the EOs’ antifungal activity, thyme, red thyme, clove, and litsea EOs inhibited the growth of the tested strains at both temperatures completely. At the same time, the growth of these strains was also inhibited by some other EOs, such as marjoram and cumin. Therefore, in the second part of the in vitro study, the lower concentration (15.625–250 μL/L of air) of these EOs (litesa, clove, cumin, thyme, red thyme, and marjoram) was tested. However, for the determination of the minimum inhibitory doses (MIDs), only those EOs (listed above) were selected that completely inhibited the growth of the tested strains during 14 days of cultivation at 25 ± 1 °C due to the better growth of the tested strains at a higher cultivation temperature. The results are summarized in Table 2.

*P. commune* KMi–183 was evaluated as the most resistant to the inhibitory effect of the tested EOs in this study. The best MID value for this strain was determined after treatment by litsea (15.62 μL/L), followed by clove (31.25 μL/L) > thyme (62.5 μL/L) > red thyme (125 μL/L) > cumin, and marjoram EOs (250 μL/L). On the contrary, the *P. commune* KMi–402 strain was evaluated as the most sensitive because it was inhibited by the doses of EOs with the best MID value for clove and litsea (less than the last tested concentration of 15.62 µL/L) > thyme (15.62 µL/L) > cumin and red thyme (62.5 µL/L) > and marjoram EOs (125 µL/L). Litsea and clove EOs were the most effective EOs from all tested EOs. These EOs showed the greatest inhibitory effect on the growth of the tested strains at a concentration equal to, or lower than, the last tested for litsea 15.62 µL/L and a concentration from 15.62 to 31.5 µL/L for clove EOs. The excellent inhibitory effect of clove and litsea EOs at lower concentrations against some strains of the genus *Penicillium* and *Aspergillus* was also confirmed in other studies [42,43].

### 3.2. Analyses of the Essential Oils

The chemical composition and percentage representation of the main components (Table 3) of the tested EOs were determined by gas chromatography with mass spectrometry (GC–MS), and gas chromatography with flame ionization detector (GC–FID). The tested EOs used in this study belong to four different plant families (Apiaceae, Lamiaceae, Myrtaceae, and Laureaceae). All EOs belonging to the Apiaceae family (fennel, anise, and cumin EOs) showed relatively significant antifungal effects, depending on the strain tested and the cultivation temperature. The results of the analysis (GC–MS and GC–MS FID) show that the EOs with the highest representation of trans anethole had the greatest antifungal effect on the growth of both tested strains: fennel (79.92%), anise (93.30%), and cumin (55.06%) EOs. Our results correspond to results of other authors, who found that cumin and anise EOs showed very good inhibitory effects on the growth of tested fungi. These authors also reported a similar composition of the main components in the used EOs [33,44]. The excellent inhibitory effect of cumin EO (*Carum carvi* L.) was also found in the study of Romagnoli et al. [45]. At the same time, the authors also analyzed the chemical composition of cumin EO using GS–MS and they found the presence of ρ-cymene as one of the main components in a higher amount (22.70%), similarly to our study (22.70% of p–cymene in cumin EO). EOs belonging to the Lamiaceae family (thyme, red thyme, rosemary, bergamot mint, sage, marjoram, and basil EOs) showed different antifungal effects on the growth of tested strains depending on their chemical composition and the content of the main components. The EOs that showed the best antifungal effect on the growth of both tested strains (100%) contained the highest levels of thymol in thyme (43.10%) and red thyme (51.51%) EOs. The main components of EOs include primarily phenolic compounds (terpenoids and phenylpropanoids), such as thymol, carvacrol, or eugenol, whose antimicrobial, antioxidant, and antifungal properties have been verified by some authors [46,47]. The EOs which did not show significant inhibitory effects compared to others from this family were those with the highest amount of estragole (88.60% in basil), geraniol (42.10% in bergamot mint), and eucalyptol (42.90% in rosemary). On the contrary, Da Silva Bomfim et al. [48] reported significant antifungal, antibacterial, or antioxidant effects of rosemary EO with the main component of eucalyptol (52.2%). In our study, EO from bergamot mint showed the lowest inhibitory effect on the growth of both tested strains. Nevertheless, some authors reported that geraniol has a very good inhibitory activity against fungi such as *Aspergillus flavus* and *A. ochraceus* [49] or *Trychophyton rubrum* [50]. However, Moleyar and Narasimham [51], who tested geraniol in vapor phase against *Aspergillus niger*, *Fusarium oxysporum*, and *Penicillium digitatum*, found that geraniol failed to completely inhibit these fungi and was more active only against *Rhizopus stolonifer* and *Mucor* sp., after adding it to liquid medium. From EOs belonging to the Myrtaceae family (clove, eucalyptus, and niaouli EOs), only clove EO with a higher proportion of eugenol (82.30%) showed significant inhibitory effects against the tested strains. Eugenol was also reported as the main component in clove EO in the study of Mulla et al. [52], and at the same time, significant antioxidant, insecticidal, and antifungal properties are attributed to it. The last analyzed EO was litsea, which belongs to the Laureaceae family. Litsea EO with β-citral (32.75%) and α-citral (40.00%) as the main compounds showed complete inhibitory effects on the growth of the tested strains. Our results agree with other authors who reported a similar composition and inhibitory effect of this EO [42,53].

### 3.3. In Vitro Inhibitory Effect of Tested Essential Oils on CPA Production

Mycotoxins can enter from feed or through the food chain into milk or dairy products. They are very often produced, for example, during cheese ripening or during storage of dairy products [54]. Due to their thermostability, their content in milk does not change significantly even by heat treatment (pasteurization). However, not all mycotoxins pose a major hazard, such as the production of CPA by *Penicillium commune* or *Penicillium camemberti* strains. However, long-term exposure to this mycotoxin present in food or feed can have various adverse effects on human or animal health. For example, Izzo et al. [55] analyzed 68 types of commercial and traditional Slovak cheeses to investigate the occurrence of fungal metabolites. They found the presence of fungal mycotoxin contamination (28 analytes) in all cheese samples, and the most often detected mycotoxin was cyclopiazonic acid (102.9%). The significant antitoxicogenic properties of EOs and their potential use for reducing or eliminating mycotoxins in food were previously confirmed [10,11,20,21,22,23]. Therefore, in this study, the antitoxicogenic activity of the tested EOs was also evaluated. For this study, only these EOs were selected which did not show a complete inhibition of *P. commune* strains growth after 14 days of cultivation at 25 ± 1 °C (eucalyptus, niaouli, fennel, basil, rosemary, sage EOs for both strains and anise and bergamot mint EOs for strain *P. commune* KMi–183) and after 35 days of cultivation at 5 ± 1 °C (eucalyptus, niaouli, fennel, basil, rosemary, and sage EOs for strain *P. commune* KMi–183 and bergamot mint EO for strain *P. commune* KMi–402). At the same time, the diameter of the grown colony had to be ≥1 cm in order to obtain the amount needed for TLC analysis.

The obtained results (Table 4) show that some EOs were not able to inhibit the growth of the tested strains completely, but they were able to suppress the production of cyclopiazonic acid. The obtained results were compared with the control sets and then expressed as the percentage of inhibition or production of mycotoxin (CPA).

The most effective EOs with a 100% antitoxicogenic effect on CPA production in both strains after 14 days of cultivation at 25 ± 1 °C were sage, basil, niaouli, and eucalyptus. Our results agree with our previous study and other authors. For example, Císarová et al. [21] tested the *in vitro* antitoxicogenic effect of some EOs (including sage, eucalyptus, and basil) on the production of secondary metabolites (aflatoxin B_1_ and aflatoxin G_1_) by thin-layer chromatography under *in vitro* conditions. Their results show a very good inhibitory effect of these EOs. The similar results with sage EO against CPA production obtained by Foltinová et al. [56] and with basil EO against aflatoxins production was reported by authors Nazzaro et al. [57]. A high inhibitory effect of CPA production in both tested strains of *P. commune* (more than 50%) in bergamot (83.33% for *P. commune* KMi–183) and rosemary (66.67% for *P. commune* Kmi–402) Eos were determined. Kedia et al. [31] reported the antitoxicogenic effect of mint EO (*Mentha spicata* L.) on the production of aflatoxin B_1_ at even lower concentrations compared to that which was effective in inhibiting the growth of the *Aspergillus flavus* strain. Other EOs, such as fennel (33.33% for strain KMi–183 and 50% for strain KMi–402) and anise (50% for both tested strain), had a lower antitoxicogenic effect on CPA production. Nevertheless, Aly et al. [58] found that anise EO was able to completely inhibit the production of aflatoxin B_1_ produced by *A. flavus*, *A. parasiticus*, and fumonisins by *Fusarium verticillioides*. However, growth of these fungi was inhibited to 85% only. In addition, Prakash et al. [59] observed the differences in the effect of tested EOs on both the growth of microscopic fungi and the production of mycotoxins.

The results of EOs’ antitoxicogenic effect in strains (KMi–183) even at a lower culture temperature (5 ± 1 °C) showed that eucalyptus EO was again the most effective, despite the fact that in the testing of antifungal activity it did not show a complete (100%) inhibitory effect on the growth of both tested strains. It may be due to the presence of limonene, one of the EO components. The authors Razzaghi-Abyaneh et al. [60] also report that those EOs that contain a high content of limonene (for example, dill, bergamot, lemon, or grapefruit EOs) did not inhibit the growth of the tested fungal strains but had the strongest inhibitory effect on the production of their secondary metabolites. Bergamot mint (66.67% for strain KMi–183 and 83.33% for strain KMi–402) and rosemary EOs (83.33% for strain KMi–183) also showed a very high inhibition effect on the production of CPA.

### 3.4. In Situ Antifungal Activity of Essential Oils on the Model Food (Cheese Samples)

A piece of Edam cheese with a fat content of 45% was chosen as the model food. To determine the minimum inhibitory doses (MIDs), the EOs were selected based on previous *in vitro* testing. The EOs with a significant inhibitory effect on mycelial growth (complete inhibition of tested strains), namely clove, thyme, red thyme, and litsea, were used. Cumin and marjoram EOs were selected for *in situ* experiments because they showed high antifungal activity against booth tested strains. Despite the fact that they did not show a complete (100%) inhibitory effect at both cultivation temperatures, they were included in the model food experiment because they could positively affect the sensory properties of the cheese. Cheese samples were inoculated with *P. commune* strains and treated with EOs by the gas diffusion method at concentrations of 250, 125, 62.5, and 31.25 μL/L. These concentrations were chosen based on the results of the MID determination in *in vitro* analyses. The effect of EOs on the growth of *P. commune* strains (KMi–183 and KMi–402) on the model food was performed after 14 and 35 days of sample storage in a refrigerator at 5 ± 1 °C.

#### 3.4.1. Determination of MID Values in *In Situ* Condition

**Figure 1:**
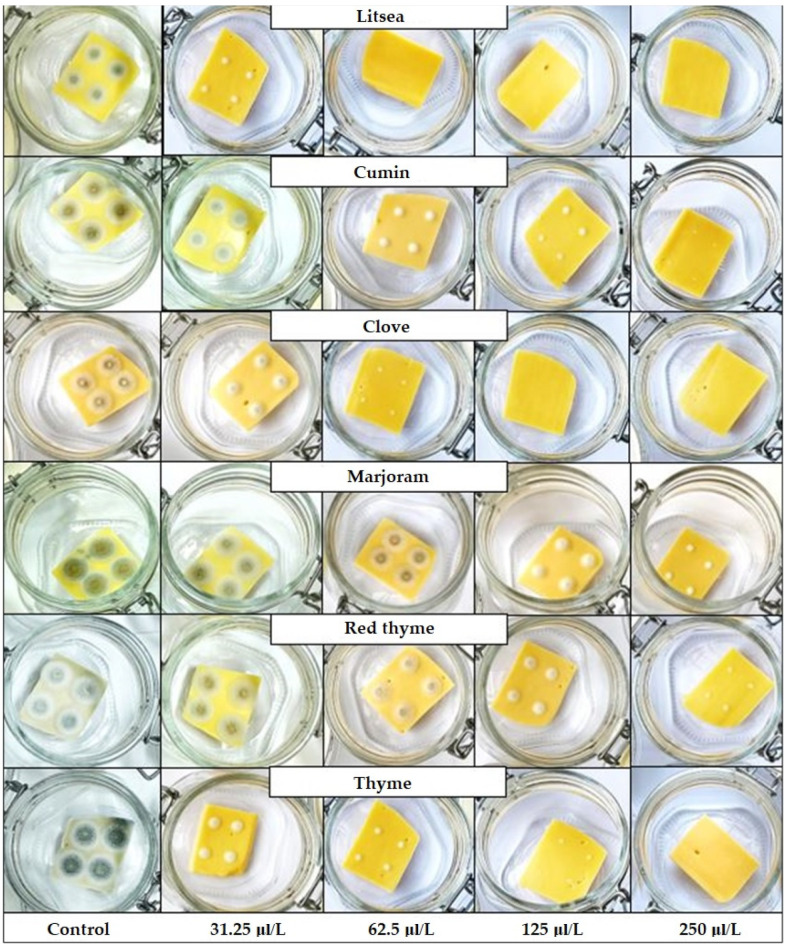
Tested concentrations (250–31.5 μL/L) of effective essential oils for the growth of *P. commune* strain (KMi–402) on model food (cheese) on the 14th day of cultivation at a temperature of 5 ± 1 °C.

According to the probity analysis, the MID_50_ and MID_90_ required to inhibit the growth of the tested *P. commune* strains were recorded (Table 5). The effect of EOs on the growth of *P. commune* strain KMi–402 after 14 days of cultivation at 5 ± 1 °C is shown in Figure 1. The sensitive strain *P. commune* KMi–402 was inhibited by the lowest inhibitory doses (MID_50_ and MID_90_) of all tested EOs on both 14th and 35th cultivation days at 5 ± 1 °C. The lowest MIDs_50_ after 14 days of cultivation for this strain was found in treatment with litsea (27.68 μL/L), followed by clove (54.20 μL/L) ˂ thyme (72.45 μL/L) ˂ red thyme (74.71 μL/L) ˂ cumin (185.76 μL/L) ˂ and marjoram (MID_50_ 285.50 μL/L). The lowest MID_90_ was found in treatment with litsea (38.17 μL/L) ˂ clove (94.63 μL/L) ˂ red thyme (107.44 μL/L) ˂ and thyme (107.7 μL/L). After 35 days of cultivation, the litsea EO reached MID_50_ values of 45.74 μL/L and MID_90_ 74.71 μL/L, and clove EO reached MID_50_ values of 66.01 μL/L and MID_90_ 76.06 μL/L. Litsea (MID_50_ 35.26 μL/L and MID_90_ 43.49 μL/L after 14 days of cultivation; MID_50_ 54.20 μL/L and MID_90_ 79.37 μL/L after 35 days of cultivation) and clove (MID_50_ 56.84 μL/L and MID_90_ 98.91 μL/L after 14 days of cultivation; MID_50_ 74.56 μL/L and MID_90_ 86.06 μL/L after 35 days of cultivation) EOs were also the most effective, with a significant growth inhibition of the *P. commune* KMi–183 strain. Excellent antifungal effects at reduced concentrations in the vapor phase of EOs were confirmed with litsea [11,29,30] and clove EOs [10,20,21,56] in previous studies.

Cumin and marjoram EOs could represent a suitable alternative to preservatives, because cumin is commonly added to some types of cheese to improve the taste (Gouda with cumin or Président Camembert cumin-ripened cheese with white mold). However, in our study, cumin EO proved to be one of the least effective EOs on the model food. The highest MIDs of cumin EOs were recorded for the strain KMi–183 MID_50_ 237.10 μL/L and MID_90_ 371.25 μL/L after 14 days of cultivation and MID_50_ 261.15 μL/L and MID_90_ 295.12 μL/L after 35 days of cultivation at 5 ± 1 °C. In our work, cumin EO was able to inhibit the growth of both tested strains of *P. commune* only at higher concentrations. Similar results were obtained by Zhaveh et al. [32], who tested the inhibitory effect of cumin EO through its encapsulation in a nanogel on the growth of some species of fungi. The lowest inhibitory doses of cumin EO for the tested fungi were recorded at a concentration of 350 μL/L, in comparison with the effect of cumin EO tested alone (without encapsulation); mycelial growth was inhibited completely only at a concentration of 650 μL/L. According to our results, marjoram EO was evaluated as the least effective from all EOs tested in this study. This EO was able to inhibit the growth of *P. commune* only up to the 14th day of cultivation, with values of MID_50_ of 292.36 μL/L (KMi–183) and MID_50_ 285.50 μL/L (KMi–402) and with MID_90_ values of 332.79 μL/L (KMi–183) and MID_90_ 324.21 μL/L (KMi–402). Marjoram EO was not able to inhibit the growth of both tested strains after 35 days of cultivation and, therefore, it was not possible to determine the MID_50_ and MID_90_ of this EO (MID > 250) using probit analysis. Similar results were obtained by Massoud et al. [61], who found that, among all tested EOs, marjoram was the least effective and was able to inhibit the growth of *Aspergillus flavus* and *Fusarium moniliforme* by only 18 to 27% at the highest tested concentration (625 μL/L). Similarly, Nedorostová et al. [62] evaluated marjoram EO as one of the least effective against tested strains of Gram-positive and Gram-negative bacteria with MID values of 530 μL/L at the highest tested concentration (530–8.3 μL/L). Our results show that EOs influenced the growth of the tested strains *in situ* (except litsea and clove) only at higher concentrations. This is related to the food matrix, as the antifungal or antibacterial effect of EOs can affect the composition of the food. A similar conclusion was observed in our previous study, where EOs also inhibited the growth of fungi from *Aspergillus* species only at the higher concentrations when they were used directly on model bread [10] or coffee beans [11]. This fact was also confirmed by other authors, who state, for example, that the physical structure of cheese can affect the distribution of EOs in the system and thereby prevent their availability to microbial cells [14,63]. Moreover, our results show that, even though the tested fungi belong to the same species and were isolated from the same commodity (Edam cheese), the tested EOs inhibited their growth with a significant difference in both *in vitro* and *in situ* conditions. Therefore, the choice of EOs and their effectiveness in food preservation depends not only on their composition, but also on the species or strain of the microscopic fungus that is inhibited by them.

According to the results of the MID evaluation in *in situ* conditions on the model food, marjoram and cumin EOs were excluded from the following experiments, because they were not able to completely inhibit the growth of the tested strains even at the highest tested concentration of 500 µL/L.

#### 3.4.2. Vitality of Lactic Acid Bacteria after Application of EOs in *In Situ* Conditions

EOs are characterized by significant antimicrobial effects on the growth of many types of bacteria and fungi. EOs are mostly used to inhibit the growth of negative or pathogenic species found in food. However, it could be assumed that they could also negatively affect the growth and metabolite production of bacteria that are intentionally used in food production. Such beneficial microorganisms include, for example, lactic acid bacteria (LABs), such as *lactobacilli* or *streptococci*, which are commonly used as part of starter cultures in cheese production [14]. Therefore, the effect of EOs on the growth of LABs in the model food was determined to exclude a negative effect on these microorganisms in this study. The effect of EOs (clove, thyme, red thyme, and litsea) in individual tested concentrations (250, 125, and 62.5 μL/L) on the growth of LABs, especially *streptococci*, in *in situ* conditions after the 14 and 35 days of cultivation were analyzed. The results are summarized in Table 6.

Our results show that almost all tested EOs (thyme, red thyme, and litsea) stimulated the growth of *Streptococcus* spp. at all tested concentrations (250, 125, and 62.5 μL/L) compared to the control sets (not treated with EOs 8.16 log CFU/g) after 14 days of cultivation. In cheeses samples treated with clove oil, a slight decrease in the vitality of *Streptococcus* spp. was noticed only in one case, at the lowest concentration (62.5 μL/L), 8.11 log CFU/g, compared to the control. The most significant stimulating effect was observed with red thyme (8.44 log CFU/g) and thyme EOs (8.42 log CFU/g) after 14 days of cultivation at a concentration of 250 μL/L. Our results agree with Mohamed et al. [64], who found that EOs used to treat cheeses (buffalo milk mozzarella) (*Anethum graveolens* L., *Carum carvi* L., *Coriandrum sativum* L., *Ocinum basilicum* L., and *Melissa officinalis* L.) had no inhibitory effect on the starter cultures of *Lactobacillus delbrueckii* subsp. *Bulgarica* and *Streptococcus thermophilus* compared to the untreated control samples. They also found that in all cases the total number of vital bacteria gradually increased until the end of storage. Burt [65] reported that LABs are resistant to the action of EOs. The vitality of *Streptococcus* spp. bacteria tested on cheeses treated with EOs stored for 35 days showed that all EOs were able to stimulate the growth of bacteria compared to the control (8.46 log CFU/g). Similarly, Marcial et al. [66] found that the tested oregano EO (*Origanum vulgare* var. Hirtum) used at concentrations of 50, 100, 150, and 250 μg/g in milk containing *S. thermophilus* (CRL 728 and CRL 813), *L. delbrueckii* subsp. *bulgaricus* (CRL 656 and CRL 468), and *Lactococcus lac*tis subsp. *lactis* (CRL 597) had no effect on cell viability, growth, acidification, and fermentation activity of the bacteria even at the highest concentration. Similarly, Sadeghi et al. [67] applied cumin essential oil (*Cuminum cyminum* L.) in concentrations of 7.5, 15, and 30 μL/mL alone and in combination with the probiotic bacteria *Lactobacillus acidophilus* (0.5% *w*/*v*) to Iranian cheese with the aim to determinate its antibacterial activity against *St. aureus* after 75 days of storage. They found that EO and probiotic bacteria had synergistic effects in reducing the growth of *St. aureus* in cheese. The greatest inhibitory activity was detected during the 75th day of storage in samples containing the highest concentration of EO (30 μL/mL) and bacteria (0.5%). In addition, cheeses treated with a concentration of 15 μL/mL achieved the highest score in the sensory evaluation. Conte et al. [68] also demonstrated that LABs are the most resistant to the effect of EOs among the Gram-positive bacteria. Moreover, LABs have antifungal and antibacterial properties and can be used as biopreservatives [69]. Our results agree with the above-mentioned authors, as the tested EOs not only did not inhibit the growth of the lactic bacteria tested (*Streptococcus* spp.) on the model food (cheese) but were able to stimulate their growth with increasing storage time compared to the control samples. Therefore, they could also be used in combination with EOs to prevent the cheese from contamination by spoilage microorganisms.

### 3.5. Sensory Analysis of Cheese Samples Treated with Selected EOs

EOs are primarily used as aromatic additives in the food industry, but their use could negatively affect the organoleptic properties of the treated food, due to their volatile compounds. For this reason, it is very important to examine how the EOs or their components react with food and how they can affect the sensory properties of specific food products [63]. Some EOs showed less inhibitory activity directly in food than in the culture medium, which is largely influenced by the properties of the food, pH, or salt concentration in the food product [14]. Therefore, it is often necessary to use higher concentrations of EOs to achieve the desired effect, which could negatively affect the sensory properties of the food product, since EOs have strong aromatic properties [70].

Therefore, in this study, the effect of EOs with a significant antifungal activity (results obtained in previous experiments) was tested on the sensory properties of the treated model food (Edam cheese) in *in situ* condition. Based on the previous results found in the determination of MIDs on a model food, the lowest tested concentration (31.5 μL/L) and non-effective EOs were excluded from the sensory evaluation. Clove, thyme, red thyme, and litsea EOs were used in three effective concentrations (250, 125, and 62.5 μL/L). The sensory evaluation was performed on the 14th and 35th day of storage (5 ± 1 °C) of a model food (cheese) treated with EOs using the triangle test. The triangle test was selected because it makes it possible to distinguish a treated sample from an untreated one without the need to specify different sensory characteristics. The obtained results are summarized in Table 7.

After 14 days of storage, the most statistically significant differences (*p* < 0.001) were found in cheese samples treated with thyme and red thyme EOs at all tested concentrations. Nevertheless, the cheeses treated with these EOs tasted and smelled pleasant and were edible. The significant differences (*p* < 0.001) were obtained for all used EOs only at the highest tested concentration (250 μL/L) in both storage periods (on the 14th and 35th day). The evaluators did not notice any significant differences (*p* ≥ 0.05) in the treated cheeses with clove and litsea EO at concentrations of 62.5 and 125 μL/L in comparison with the control samples of cheeses, in both evaluation periods (on the 14th and the 35th day). The least recognizable was clove EO at both concentrations (62.5 and 125 μL/L) on the 14th and 35th day of evaluation.

Zantar et al. [17] tested the effect of thyme and oregano EOs on some pathogenic microorganisms directly on goat cheeses. The tested EOs were added to goat cheese in concentrations of 0.05 and 0.1%, and they found that both EOs extended their shelf life. Regarding the sensory evaluation, oregano was evaluated as unpleasant in relation to the taste of the cheese. In contrast, the results of cheese treated by thyme were much more acceptable. El-Kholy et al. [71] tested the effect of EOs on the quality and shelf life of cottage cheese. They found that the cheese containing EOs (thyme, rosemary, and caraway) had an extended shelf life and acceptable sensory properties even at the end of the storage period. Nevertheless, some authors state that EOs can have undesirable effects on the taste and smell of cheese during long-term storage. Olmedo et al. [72] reported that cheeses treated with EOs changed their typical flavors the most on the last day of storage and evaluation of the cheeses. Similarly, Pettersen et al. [73] reported that they noted an increase in the bitter and sour taste in cream cheese treated with EOs incorporated in special polyethylene packages, cultured in the dark at a temperature of 5 ± 1 °C for 35 days. However, our results show that these EOs (clove, thyme, red thyme, and litsea) did not interfere with the overall evaluation of the treated cheese samples. On the contrary, cheeses treated with EOs were evaluated as tastier compared to the control. This fact may be related to the used method selected for cheese sample treatments. Our results show that the use of EOs in the vapor phase does not have an undesirable effect on the organoleptic properties of cheeses even after 35 days of storage.

## 4. Conclusions

Our results show that EOs, namely clove, thyme, red thyme, and litsea, which were tested against two isolates of *P. commune* (KMi–183 and KMi–402) *in vitro* and *in situ* (on a model food—cheese), were able to inhibit these strains. Litsea and clove EOs were evaluated as the most effective in the vapor phase, not only *in vitro*, but also *in situ* experiments in this study. Moreover, our results show that these EOs did not interfere with the overall evaluation of the treated cheese and were able to inhibit the growth of *P. commune* during 35 days of storage. EOs also did not inhibit the growth of the lactic bacteria tested (*Streptococcus* spp.) on the model food (cheese). Although the other tested EOs could not significantly inhibit the growth of the tested strains compared to the control sets, they could be used in combination with more effective EOs, for example, for their ability to suppress the production of mycotoxins (eucalyptus, sage, niaouli EOs) or to improve the sensory properties of cheeses (marjoram, cumin EOs). Therefore, combining EOs to achieve a broad effect and thereby increase the protection of food products would be advisable. Based on the results of this study, it can be concluded that the treatment of cheese (Edam cheese 45%) with some EOs could extend its shelf life and protect it from this contaminant.

## Figures and Tables

**Table 1:** *In vitro* inhibitory effects of essential oils at 25 ± 1 and 5 ± 1 °C after 14 days of cultivation and 35 days of cultivation on growth of *Penicillium commune* strains (KMi–183 and KMi–402) (PDs divided into three sectors in independent repetition were used (*n* = 6)).

Tested Essential Oils	*P. commune* (KMi–183) *				*P. commune* KMi–402			
	Days of Cultivation							
	14 Days	35 Days	MGI (%)		14 Days	35 Days	MGI (%)	
	Mean Colony Diameter in cm ± SD		14th Days	35th Days	Mean Colony Diameter in cm ± SD		14th Days	35th Days
C/T/RT/L	0.00 ± 0.00 ^a^	0.00 ± 0.00 ^a^	100.00	100.00	0.00 ± 0.00 ^a^	0.00 ± 0.00 ^a^	100.00	100.00
Eucalyptus	4.16 ± 1.07 ^ef^	1.65 ± 0.05 ^de^	2.80	16.08	3.40 ± 0.13 ^h^	0.95 ± 0.51 ^cd^	20.39	36.34
Niaouli	2.71 ± 1.06 ^cde^	0.60 ± 0.10 ^bc^	33.79	69.48	1.63 ± 0.16 ^cd^	0.53 ± 0.04 ^b^	61.83	64.57
Fennel	2.58 ± 0.21 ^cd^	0.38 ± 0.20 ^b^	37.02	80.54	1.79 ± 0.11 ^de^	0.00 ± 0.00 ^a^	57.97	100.00
Anise	3.06 ± 1.03 ^cdef^	0.00 ± 0.00 ^a^	25.17	100.00	1.22 ± 0.06 ^b^	0.00 ± 0.00 ^a^	71.37	100.00
Cumin	0.51 ± 0.78 ^ab^	0.00 ± 0.00 ^a^	87.46	100.00	0.00 ± 0.00 ^a^	0.00 ± 0.00 ^a^	100.00	100.00
Basil	2.73 ± 1.05 ^cde^	0.82 ± 0.12 ^c^	33.36	58.29	2.16 ± 0.16 ^f^	0.42 ± 0.13 ^b^	49.27	71.92
Rosemary	3.44 ± 1.56 ^def^	1.34 ± 0.45 ^d^	16.07	32.10	1.93 ± 0.18 ^e^	0.56 ± 0.31 ^bc^	54.83	62.57
Sage	1.77 ± 0.83 ^bc^	0.64 ± 0.03 ^bc^	56.75	67.70	1.57 ± 0.09 ^c^	0.00 ± 0.00 ^a^	63.18	100.00
BM	3.39 ± 0.02 ^def^	1.87 ± 0.31 ^e^	17.30	4.51	2.83 ± 0.09 ^g^	1.32 ± 0.45 ^de^	33.67	11.77
Marjoram	0.75 ± 0.27 ^ab^	0.00 ± 0.00 ^a^	81.71	100.00	0.00 ± 0.00 ^a^	0.00 ± 0.00 ^a^	100.00	100.00
Control	4.31 ± 0.71 ^f^	1.97 ± 0.05 ^f^	-	-	4.26 ± 0.03 ^ch^	1.50 ± 0.04 ^e^	-	-

Data in the column followed by different letters are significantly different in 95.0% Tukey HSD test, *p* < 0.05. *—strain ID, C—Clove, RT—Red thyme, T—Thyme, L—Litsea, BM—bergamot mint, SD—Standard deviation, MGI (%)—mycelial growth inhibition in %.

**Table 2:** Minimum inhibitory doses (MIDs) of effective essential oils able to inhibit growth of *Penicillium* strains at 25 ± 1 °C after 14 days of cultivation (PDs divided into three sectors in two independent repetition were used (*n* = 6)).

Tested Essential Oils	MIDs (µL/L of Air) of EOs	
	*Penicillium commune* (KMi–183) *	*Penicillium commune* (KMi–402)
Clove	31.25	<15.625
Thyme	62.5	15.625
Red thyme	125	62.5
Litsea	15.625	<15.625
Cumin	250	62.5
Marjoram	250	125

*—strain ID.

**Table 3:** The chemical composition and quantification of the major components in tested essential oils.

RI ^b^		Component	C ^c^	T	RT	L	E	N	F	A	Cu.	B	R	S	BM	M
932		α-Thujene									6.50			0.40		
938	^a^	α-Pinene	0.15	2.50	1.00	1.56	2.70	9.80	2.11	0.70	2.40	0.26	8.57	5.10		1.50
953	^a^	Camphene		1.52	1.21				0.10				4.08	6.09		
980	^a^	β-Pinene		0.12	1.50	1.12	0.40	2.30	0.23			0.30	8.19	3.00		5.40
993		β-Myrcene		1.36											1.00	
1006	^a^	α-Phellandrene					0.50									
1019	^a^	α-Terpinene			0.80		0.10									8.00
1029	^a^	p-Cymene		39.10	16.51		6.30	1.99			22.70		3.00	1.80	0.30	4.60
1031	^a^	D-Limonene		0.86		13.66	6.90	6.80	5.11		9.70	0.25	2.60	1.98	1.13	3.40
1032		β-Phellandrene				2.60										
1034	^a^	Eucalyptol	0.30	1.47	1.30	3.60	79.30	54.30	0.10			3.98	42.90	11.00	0.50	
1062	^a^	γ-Terpinene			4.60		2.71		0.10		1.00		0.77	1.10		13.10
1071		5-Isopropyl-2-methylbicyclo[3.1.0]hexan-2-ol														4.60
1090	^a^	Terpinolene					0.12		4.52				2.22	0.55		3.60
1101	^a^	Linalool		5.10	5.20	1.13	0.86			0.50		1.53	0.53	0.39	37.20	14.20
1108		α-Thujone												23.00		
1119		β-Thujone												6.46		
1122		β-Terpinene									1.76					
1147	^a^	(-)-Isopulegol			1.70				0.11			0.33	13.10	20.11		1.00
1158	^a^	(+/−)-citronellal				0.77										
1168	^a^	Borneol		1.85	1.66	0.89							3.80	4.20		
1179	^a^	4-Terpineol											0.46	0.50		
1181	^a^	(+/−)-Menthol			1.60								0.50		2.80	30.70
1186		cis-Verbenol				1.00										
1192		α-Terpineol		1.10				9.21				0.20	2.27		1.30	3.60
1199		4-Allylanisole							4.40	2.75			0.30			
1202	^a^	Estragol										88.60				
1238		Thymol methyl ether			0.50											
1245	^a^	β-Citral				32.70										
1247	^a^	(-)-carvone			1.87						55.06					
1259	^a^	Geraniol				0.96			1.15	1.45					42.10	2.10
1275		α-Citral				40.00										
1287		Bornyl acetate										0.16	1.31	3.37		
1289		Anethole							79.92	93.30						
1296	^a^	Thymol		43.10	51.51											
1306	^a^	Carvacrol		0.70	3.00						0.66					
1352		α-Terpineol acetate						1.00								
1360	^a^	Eugenol	82.30													
1368		Neryl acetate													2.30	
1386	^a^	Geranyl acetate							1.26	0.30					7.20	
1403		(+)-Longifolene											0.22			
1407		Eugenol methyl ether										0.30				
1420	^a^	Caryophyllene	6.00	0.90	5.10			1.40				0.14	3.60	7.38	2.35	2.40
1435		α-Bergamotene										2.28				
1452	^a^	α-Caryophyllene	2.60										0.33	4.30		
1478		Germacrene D										0.73				
1481		α-Curcumene										0.14				
1496		Elixene														0.80
1510		γ-Cadinene										0.45				
1531		Eugenol acetate	7.85													
1567		.±-trans-Nerolidol						2.30								
1574	^a^	Caryophyllene oxide		0.19	0.50								0.30			
1592		Viridiflorol						10.50								
		total	99.20	99.87	99.56	99.99	99.89	99.60	99.11	99.00	99.85	99.65	99.05	99.73	99.18	99.00

^a^ Identification confirmed by co-injection of authentic standard. ^b^ RI: identification based on Kovat’s retention indices (HP-5MS capillary column) and mass spectra. ^c^ Relative proportions were calculated in % by dividing individual peak area by total area of all peaks. C—clove, T—thyme, RT—red thyme, L—litsea, E—eucalyptus, N—niaouli, A—anise, Cu.—cumin, B—basil, R—rosemary, S—sage, BM—bergamot mint, M—marjoram.

**Table 4:** *In vitro* inhibitory effects (in %) of essential oils (500 µL/L of air) at 25 ± 1 °C after 14 days of cultivation and at 5 ± 1 °C after 35 days of cultivation on the mycotoxin production by *Penicillium commune* strains (PDs divided into three sectors in two independent repetition were used (*n* = 6)).

Essential Oils	Tested Temp./Cult. Days	*Penicillium commune* (KMi–183) *		*Penicillium commune* (KMi–402)	
		Production of CPA (%)	Inhibition of CPA (%)	Production of CPA (%)	Inhibition of CPA (%)
Eucalyptus	5 ± 1 °C/35 d	0	100	NA	NA
	25 ± 1 °C/14 d	0	100	0	100
Niaouli	5 ± 1 °C/35 d	NA	NA	NA	NA
	25 ± 1 °C/14 d	0	100	0	100
Fennel	5 ± 1 °C/35 d	NA	NA	NA	NA
	25 ± 1 °C/14 d	66.67	33.33	50.00	50.00
Anise	5 ± 1 °C/35 d	NA	NA	NA	NA
	25 ± 1 °C/14 d	50.00	50.00	50.00	50.00
Basil	5 ± 1 °C/35 d	NA	NA	NA	NA
	25 ± 1 °C/14 d	0	100	0	100
Rosemary	5 ± 1 °C/35 d	16.67	83.33	NA	NA
	25 ± 1 °C/14 d	33.33	66.67	33.33	66.67
Sage	5 ± 1 °C/35 d	NA	NA	NA	NA
	25 ± 1 °C/14 d	0	100	0	100
Bergamot mint	5 ± 1 °C/35 d	33.33	66.67	16.67	83.33
	25 ± 1 °C/14 d	16.67	83.33	0	100
Control	5 ± 1 °C/35 d	100	0	100	0
	25 ± 1 °C/14 d	100	0	100	0

*—strain ID, Temp.—Temperature, Cult.—cultivation, CPA—cyclopiazonic acid, NA—not analyzed. d—day.

**Table 5:** Minimum inhibitory doses (MID_50_ and MID_90_) for used essential oils able to inhibit growth of *Penicillium commune* strains on cheese samples at 5 ± 1 °C (three replications in treatments with each essential oil were screened (four colonies in three repetition (*n* = 12)) after 14 and 35 days of cultivation.

Essential Oils	MID (μL/L of Air)	Tested Strains			
		*Penicillium commune* (KMi–183) *		*Penicillium commune* (KMi–402)	
		14th Days	35th Days	14th Days	35th Days
Clove	MID_50_	56.84	74.56	54.30	66.01
	MID_90_	98.91	86.06	94.63	76.06
Thyme	MID_50_	103.27	133.95	72.45	120.23
	MID_90_	140.36	151.75	107.72	134.75
Red thyme	MID_50_	125.00	142.86	74.71	88.51
	MID_90_	140.64	162.38	107.44	126.17
Litsea	MID_50_	35.26	54.20	27.68	45.74
	MID_90_	43.49	79.37	38.17	74.71
Cumin	MID_50_	237.10	261.15	185.76	250.00
	MID_90_	371.25	295.12	306.53	281.26
Marjoram	MID_50_	292.36	>250	285.50	>250
	MID_90_	332.79	>250	324.21	>250

MID_50_—concentration causing 50% reduction in mycelial growth, MID_90_—concentration causing 90% reduction in mycelial growth. *—strain ID.

**Table 6:** The number of viable cells (log CFU/g) of the *Streptococcus* spp. in a model food (cheese) treated with essential oils on the 14th and 35th days of storage at a temperature of 5 ± 1 °C.

Tested EOs	Tested Concentrations (μL/L)	log CFU/g	
		*Streptococcus* spp.	
		14 Days	35 Days
**Clove**	250	8.40	9.26
	125	8.30	9.23
	62.5	8.11	9.18
**Thyme**	250	8.42	9.69
	125	8.38	9.46
	62.5	8.30	9.27
**Red thyme**	250	8.44	9.30
	125	8.37	8.46
	62.5	8.34	8.30
**Litsea**	250	8.41	9.26
	125	8.36	9.20
	62.5	8.35	9.00
Control *		8.16	8.46

*—without essential oil treatment.

**Table 7:** Sensory evaluation of the cheese samples treated by the most effective essential oils on 14th and 35th day after storage at 5 ± 1 °C in refrigerator.

Essential Oils	Conc. µL/L	14th Day		35th Day	
		Correct Replies (%)	*p*-Value	Correct Replies (%)	*p*-Value
Clove	62.5	9 (20.00%) ^ns^	0.058	9 (20.00%) ^ns^	0.058
	125	10 (22.22%) ^ns^	0.153	12 (26.66%) ^ns^	0.429
	250	33 (73.33%)	0.001	28 (62.22%)	0.001
Thyme	62.5	29 (64.44%)	0.001	15 (33.33%) ^ns^	1
	125	30 (71.11%)	0.001	12 (26.66%) ^ns^	0.429
	250	33 (73.33%)	0.001	30 (71.11%)	0.001
Red thyme	62.5	24 (53.34%)	0.001	12 (26.66%) ^ns^	0.429
	125	30 (66.67%)	0.001	15 (33.33%) ^ns^	1
	250	33 (73.33%)	0.001	29 (64.44%)	0.001
Litsea	62.5	11 (24.45%) ^ns^	0.268	12 (26.66%) ^ns^	0.429
	125	14 (31.12%) ^ns^	0.874	13 (28.88%) ^ns^	0.635
	250	27 (60.00%)	0.001	33 (73.33%)	0.001

conc.—concentration, ns—Not significantly different. For *n* = 45, the difference between samples would be significant if the number of correct answers was 24 or more (*p* ≤ 0.01), ISO Standards 4120:2004, *p*-value—calculated according the correct and incorrect answers by the chi-squared binomial distribution with value of α-risk = 0.05.

## Data Availability

All related data and methods are presented in this paper. Additional inquiries should be addressed to the corresponding author.
